# Evaluation of perinatal outcome for growth restricted fetuses

**DOI:** 10.6026/9732063002001378

**Published:** 2024-10-31

**Authors:** Neha Hajare, Anjali Patil, Sanjaykumar Patil

**Affiliations:** 1 Department of Obstetrics and Gynaecology, Krishna Institute of Medical Sciences, Karad - 415110, Maharashtra, India

**Keywords:** Doppler, Perinatal Outcomes, abnormalities, abnormal perinatal outcomes, middle cerebral artery, umbilical artery, pulsatility index, middle cerebral artery and systolic/diastolic, growth restricted, pregnancy management

## Abstract

The usefulness of Doppler abnormalities in predicting abnormal perinatal outcomes is known. Therefore, it is of interest to assess perinatal outcomes using the fetal growth restriction and gestational age time of delivery. Hence, a comprehensive review of prenatal history and past events on 94 cases was completed using estimated fetal weight to make the fetal growth restriction diagnosis. Thus, the need for prompt treatments to improve perinatal outcomes using Doppler monitoring in growth restricted pregnancy management is highlighted.

## Background:

Intrauterine growth restriction (IUGR), also known as fetal growth restriction (FGR), is defined by an estimated fetal weight (FW) or abdominal circumference less than the 10th percentile for gestational age [1, 2]. Certain fetuses are very small, yet their risk of perinatal illness and mortality (MT) is the same. Fetuses with growth constraints, regardless of the degree of divergence from their GA, are at a higher risk of death [2, 3]. To offer appropriate therapy, it is critical to identify FGR who are at high risk of complications. Doppler ultrasonography (DP-U/S) is used to diagnose IUGR fetuses (to differentiate between small-for-dates and FGR) as well as to track sickness progression in utero [4]. The umbilical artery (UA) and vein are the most extensively studied and used vascular, followed by the middle cerebral artery (MCA) [5]. The systolic/diastolic (S/D) ratio, resistance index (RI), and Pulsatility index (PI) are the three most commonly used DP indicators used to assess arterial (AR) B/F resistance and detect IUGR [4, 6, 7]. Around 3 to 10% of pregnancies experience IUGR. Every year, around 30 million infants experience IUGR [8]. Based on data from the National Neonatal (NN) Perinatal Database of India, it has been found that approximately 9.65% of infants born in hospitals experience IUGR [9]. Ensuring proper monitoring of pregnancy with IUGR complications is crucial for enhancing the well-being of the fetus. These tests involve Cardiotocography (CTG), serial fetal biometry (SFB) measurements with fetal biophysical profiles, and color Doppler (CD) studies of utero-placental (UTP) and feto-placental circulation (FPC). They play a crucial role in assessing the oxygen levels of the fetus and ensuring prompt intervention for high-risk (HR) pregnancy. Colour DP studies have shown to be highly effective in identifying life-threatening complications at an early stage, enabling prompt decisions regarding pregnancy termination [10]. The PN-MT rates in growth restricted -NN are 6 to 10 times greater than those in NN with normal development [2]. Several studies have shown that newborns who are not allowed to grow have a higher chance of getting respiratory distress syndrome (RDS), necrotizing enterocolitis (NEC), intra-ventricular hemorrhage (IVH), coagulation disorders (CGD) and failure of multiple organs(FOMO) [2, 7]. Absence or reversal of end-diastolic flow velocities (EDFV) in the umbilical arteries (UB-A) has been associated with elevated Perinatal death rates [2, 11, 12, 13]. Therefore, it is of interest to report the relation between delivery time interval in fetal growth restriction and gestational age to predict the perinatal outcome.

## Materials and Methods:

The current prospective observational clinical study conducted over a period of one and a half years in the Department of Obstetrics and Gynecology with total of 94 patients. Antenatal history and previous events was recorded using a predetermined proforma and history taking. FGR diagnosis was based on EFW or AC <10th percentile for GA. DP studies of UM-A, and MCA was performed. GA at FGR diagnosis and delivery was noted. Antenatal steroid administration (ATS-A) was given as per hospital guidelines. Data on maternal age(MA), domicile, parity, morbidities, socio demographic (SDG) details, infertility treatment(IF-T), previous obstetric history(P-OB-h/o), GA at diagnosis and delivery, mode of delivery (including indications for caesarean section(CS)) was collected. Patient underwent serial DP assessments. DP prior to delivery (DV) (deciding DP) will be evaluated for perinatal outcomes and mode of DV. Patients were monitored and assessed with CTG and DP velocimetry (VM).

## Inclusion criteria:

All singleton pregnancy with FGR diagnosed via ultrasonography criteria of estimated fetal weight (FW) or Abdominal Circumference (AC) <10th percentile for GA, delivering in our hospital, irrespective of GA and maternal risk factors (M-R/F).

## Exclusion criteria:

[1] Anomalous Babies

[2] Multifetal pregnancy

## Statistical analysis:

Descriptive statistics was summarising the maternal (MT), fetal and perinatal characteristics. Comparative analysis was performed to assess the correlation between DP parameters and perinatal outcomes.

## Results:

Table 1 shows that, among 37 cases with an abnormal UA systolic/diastolic Ratio, 28 (57.1%) experienced adverse outcomes, significantly higher than the 9 (20.0%) with good outcomes (p < 0.001). In contrast, among 57 cases with a normal UA systolic/diastolic Ratio, 21 (42.9%) had adverse outcomes, while 36 (80.0%) had good outcomes. Therefore, found statistically significant association as the p value was <0.001. Table 2 shows that, among 20 cases with abnormal UA RI, 16 (32.7%) experienced adverse outcomes, significantly higher than the 4 (8.9%) with good outcomes (p = 0.004). Conversely, among 74 cases with normal UA RI, 33 (67.3%) cases had adverse outcomes, while 41 (91.1%) cases had good outcomes. Therefore, found statistically significant association as the p value was 0.004. Table 3 shows that, among 21 cases with abnormal UA PI, 18 (36.7%) cases experienced adverse outcomes, significantly higher than the 3 (6.7%) cases with good outcomes (p < 0.001). Conversely, among 73 cases with normal UA PI, 31 (63.3%) had adverse outcomes, while 42 (93.3%) had good outcomes. Therefore, found statistically significant association as the p value was <0.001. Table 4 shows that, among cases with an abnormal MCA systolic/diastolic Ratio (n=24), 42.9% (21 cases) experienced adverse outcomes, significantly higher than the 6.7% (3 cases) with good outcomes (p < 0.001). Conversely, among cases with a normal MCA systolic/diastolic Ratio (n=70), 57.1% (28 cases) had adverse outcomes, while 93.3% (42 cases) had good outcomes. Therefore, found statistically significant association as the p value was <0.001.

Table 5 shows that, among cases with abnormal MCA RI, 2 (4.1%) experienced adverse outcomes, with no cases showing good outcomes. In contrast, among 92 cases with normal MCA RI, 47 (95.9%) had adverse outcomes, while all cases (100.0%, 45 cases) had good outcomes. Table 6 shows that, among cases with abnormal MCA PI (n=10), 9 (18.4%) experienced adverse outcomes, significantly higher than the 1 (2.2%) with good outcomes (p = 0.011). In contrast, among 84 cases with normal MCA PI, 40 (81.6%) had adverse outcomes, while 44 (97.8%) had good outcomes. Therefore, found statistically non-significant association as the p value was 0.011. Table 7 shows that, among 27 cases with an abnormal MCA/UA PI ratio, 23 (46.9%) experienced adverse outcomes, significantly higher than the 4 (8.9%) with good outcomes (p < 0.001). Conversely, among 67 cases with a normal MCA/UA PI ratio, 26 (53.1%) had adverse outcomes, while 41 (91.1%) had good outcomes. Therefore, found statistically significant association as the p value was <0.001. Table 8 shows that, the proportion of adverse outcomes was highest in >37 weeks group 40 (81.6%), though it also had the highest number of good outcomes 30 (66.7%). Deliveries between 33-36 weeks had a higher percentage of good outcomes 14 (31.1%) compared to adverse outcomes 4 (8.2%). P value <0.001 (Significant) indicating that cases with good perinatal outcome were significantly higher in 33-36 weeks of GA compared to others

## Discussion:

Late-onset FGR occurs when a fetus fails to reach its full developmental potential after 32 weeks of pregnancy. Although it has fewer prenatal complications than early-onset FGR, it has a higher chance of negative short- and long-term consequences, including hypoxemic episodes and minor neurodevelopmental impairments, as compared to typically growing fetuses [14, 15-16]. In the study by Gaikwad *et al.* UA, the Systolic to Diastolic (S/D) ratio identified abnormalities (AB-N) in 77.6% of cases, with 14.5% classified incorrectly as abnormal (false positives). Meanwhile, it missed AB-N in 32.9% of cases (false negatives). UA Resistance Index (RI) showed AB-N in 81.5% of cases, with a false positive rate (FPR) of 5.1%, and missed AB-N in 41.8% of cases. UA-PI identified AB-N in 85.7% of cases, with a FPR of 4.1%, and missed AB-N in 40.2% of cases. Conversely, MCA indices demonstrated higher accuracy, particularly in systolic/diastolic ratio (87.5%) and PI (92.3%), with lower FPR across the board, indicating their potential for robust assessment of fetal health (FH) during pregnancy. In present study the UA parameters show that the systolic/diastolic Ratio was normal in 57 (60.6%) and abnormal (AB) in 37 (39.4%). The RI & PI of UA were normal in 74 (78.7%) and 73 (77.7%), respectively, with AB-R of 20 (21.3%) and 21 (22.3%), respectively. For MCA, the SD Ratio was normal in 70 (74.5%) and AB in 24 (25.5%). Similarly, the RI and PI of MCA were predominantly normal at 92 (97.9%) and 84 (89.4%), respectively, with AB-R of 2 (2.1%) and 10 (10.6%), respectively. The PI ratio of MCA to UA showed normal findings in 67 (71.3%) and AB findings in 27 (28.7%). Moreover, among the 94 cases analyzed, 45 (47.9%) had a good perinatal outcomes, while 49 (52.1%) experienced adverse (EA) perinatal outcomes. Specifically, 21 (42.9%) required lower segment cesarean section (LSCS) due to FD, and 35 (71.4%) had meconium-stained liquor (MSL). A significant proportion, 30 (61.2%), had an APGAR score at 5 minutes below 7, and 44 (89.8%) required admission to the NN-ICU. However, PN death occurred in 5 (10.2%) of cases. In present study the sensitivity of the UA systolic/diastolic ratio is 57.14%, with a 95% CI ranging from 42.21% to 71.18%. Specificity is notably higher at 80.00%, with a narrower CI of 65.40% to 90.42%. The Positive Predictive Value (PPV) stands at 75.68%, suggesting its reliability in identifying cases with adverse outcomes, while the Negative Predictive Value (NPV) is 63.16%, indicating its effectiveness in ruling out AO. Overall accuracy is 68.09%, encompassing the ratio's comprehensive utility in assessing FH during pregnancy. Below are compare studies listed in Table 9.

In present study among 20 cases with abnormal UA RI, 16 (32.7%) EAO, significantly higher than the 4 (8.9%) with good outcomes (GO) (p= 0.004). Conversely, among 74 cases with normal UA RI, 33 (67.3%) cases had AO, while 41 (91.1%) cases had GO. In present study sensitivity of UA RI is 32.65%, with a 95% confidence interval (CI) ranging from 19.95% to 47.54%. Specificity is notably higher at 91.11%, with a CI of 78.78% to 97.52%, indicating its ability to accurately identify cases with GO. The PPV stands at 80.00%, while the NPV is 55.41%. Overall accuracy is 60.64%, encompassing the index's comprehensive utility in assessing FH during pregnancy. Below are some of the studies which were comparable to our studies as shown in Table 10. 

In present study among 21 cases with abnormal UA PI, 18 (36.7%) cases experienced adverse outcomes, significantly higher than the 3 (6.7%) cases with good outcomes (p < 0.001). Conversely, among 73 cases with normal UA PI, 31 (63.3%) had adverse outcomes, while 42 (93.3%) had good outcomes. In addition to this, the sensitivity of the MCA systolic/diastolic Ratio is 42.86%, with a 95% confidence interval (CI) ranging from 28.82% to 57.79%. Specificity is notably higher at 93.33%, with a CI of 81.73% to 98.60%, indicating its ability to accurately identify cases with good outcomes. The Positive Predictive Value (PPV) stands at 87.50%, while the Negative Predictive Value (NPV) is 60.00%. Overall accuracy is 67.02%, encompassing the ratio's comprehensive utility in assessing FH during pregnancy. Our data are almost similar to study done by Biswas *et al.* [20] Khanduri *et al.* [21] Singh *et al.* [22] as shown in Table 11.

In present study among cases with AB-MCA RI, 2 (4.1%) EAO, with no cases showing GO. In contrast, among 92 cases with normal MCA RI, 47 (95.9%) had AO, while all cases (100.0%, 45 cases) had GO. Moreover, the sensitivity of MCA RI is notably low at 4.08%, with a wide 95% confidence interval (CI) ranging from 0.50% to 13.98%. Specificity, however, is 100.00%, with a CI of 92.13% to 100.00%, indicating its ability to accurately identify cases with GO. The PPV was 100.00%, although with a wide CI from 15.81% to 100.00%. The NPV is 48.91%. Overall accuracy is 50.00%, showing the mixed diagnostic performance of MCA RI in assessing FH during pregnancy, largely attributed to its very low sensitivity (LS). Sensitivity of MCA RI in our study is very low to compare with other studies such as Sachin *et al.* [21] and Singh *et al.* [22] However, specificity, NPV and PPV value is almost similar and comparable as shown in Table 12.

In present study the sensitivity of MCA PI is 18.37%, with a 95% confidence interval (CI) ranging from 8.76% to 32.02%. Specificity is notably high at 97.78%, with a CI of 88.23% to 99.94%, indicating its ability to accurately identify cases with GO. The PPV stands at 90.00%, suggesting strong reliability in predicting AO when MCA PI is abnormal, with a CI from 54.27% to 98.56%. The NPV is 52.38%, indicating moderate effectiveness in ruling out AO, with a CI from 48.88% to 55.85%. Overall accuracy is 56.38%, showing the mixed diagnostic performance of MCA PI in assessing FH during pregnancy, mainly due to its LS. The MCA PI association with unfavorable perinatal outcomes was compared to previous research. The Gaikwad *et al.* demonstrated a statistically significant connection between MCA PI and pregnancy outcome (p<0.05) [10]. The specificity and PPV of MCA PI in the investigation are 98.39% and 92.31%, respectively as shown in Table 13. Fetal growth restriction and small for gestational age (SGA) were associated with unfavorable perinatal outcomes. We used the categorization of fetal growth restriction at diagnosis as an independent variable to predict respiratory distress and the need for neonatal resuscitation. The model that integrates both fetal growth restriction (FGR) classification and gestational age at birth accurately predicts the need for NICU admission.

## Conclusion:

Data shows a U-shaped curve in adverse outcome relative to gestational age, highlighting the increased risks in preterm and post-term deliveries. Adverse outcome were significantly higher in 37 weeks. Therefore, it is important to timely diagnose and monitor the patient. Thus, timely delivery is crucial in intra-uterine growth restriction cases to improve outcomes.

## Figures and Tables

**Table 1:** Ratio between UAS/D for outcomes

**UA S/D Ratio**	**Adverse Outcome**		**Good Outcome**		**P value**
	**Cases**	**%**	**Cases**	**%**	
Abnormal (n=37)	28	57.10%	9	20.00%	< 0.001
Normal (n=57)	21	42.90%	36	80.00%	
Total	49	100%	45	100.00%	

**Table 2:** UA, resistance index (RI) & PGO

**UA R.I.**	**Adverse Outcome**		**Good Outcome**		**P value**
	**Cases**	**%**	**Cases**	**%**	
Abnormal (n= 20)	16	32.70%	4	8.90%	0.004
Normal (n=74)	33	67.30%	41	91.10%	
Total	49	100%	45	100.00%	

**Table 3:** UA, Pulsatility Index (PI) and PGO

**UA P.I.**	**Adverse Outcome**		**Good Outcome**		**P value**
	**Cases**	**%**	**Cases**	**%**	
Abnormal (n=21)	18	36.70%	3	6.70%	lt; 0.01
Normal (n=73)	31	63.30%	42	93.30%	
Total	49	100%	45	100.00%	

**Table 4:** Middle aerebral artery (MCA) Systolic to Diastolic (S/D) Ratio and PGO

**MCA S/D Ratio**	**Adverse Outcome**		**Good Outcome**		**P value**
	**Cases**	**%**	**Cases**	**%**	
Abnormal (n=24)	21	42.90%	3	6.70%	< 0.001
Normal (n=70)	28	57.10%	42	93.30%	
Total	49	100%	45	100.00%	

**Table 5:** MCA-RI & PGO

**MCA R.I.**	**Adverse Outcome**		**Good Outcome**		**P value**
	**Cases**	**%**	**Cases**	**%**	
Abnormal (n=02)	2	4.10%	0	0.00%	-
Normal (n=92)	47	95.90%	45	100.00%	
Total	49	100%	45	100.00%	

**Table 6:** MCA-PI & PGO

**MCA PI**	**Adverse Outcome**		**Good Outcome**		**P value**
	**Cases**	**%**	**Cases**	**%**	
Abnormal (n=10)	9	18.40%	1	2.20%	0.011
Normal (n=84)	40	81.60%	44	97.80%	
Total	49	100%	45	100%	

**Table 7:** MCA/UA- PI ratio

**MCA/UA P.I. Ratio**	**Adverse Outcome**		**Good Outcome**		**P value**
	**Cases**	**%**	**Cases**	**%**	
Abnormal (n=27)	23	46.90%	4	8.90%	< 0.01
Normal (n=67)	26	53.10%	41	91.10%	
Total	49	100%	45	100%	

**Table 8:** Gestational Age (GA) distribution

**Gestational age(GA)**	**Adverse Outcome**		**Good Outcome**		**Total**
	**Cases**	**%**	**Cases**	**%**	
28-32 weeks	5	10.20%	1	2.20%	6
33-36 weeks	4	8.20%	14	31.10%	18
>37 weeks	40	81.60%	30	66.70%	70
Total	49	100.00%	45	100.00%	94

**Table 9:** UA-S/D ratio with other studies

**Various Study**	**Sensitivity**	**Specificity**	**PPV**	**NPV**	**Diagnostic accuracy**
Lakhkar *et al.* [16]	66.60%	45.40%	66.60%	45.40%	-
Netam *et al.* [17]	86.96%	71%	51.28%	94%	75%
Purushotham *et al.* [18]	83.30%	93.70%	88.20%	90.90%	-
Gaikwad *et al.*[19]	60.32%	82.26%	77.55%	67.11%	71.20%
Present study	57.14%	80.00%	75.68%	63.16%	68.09%

**Table 10:** UA-RI with other studies

**Various Study**	**Sensitivity**	**Specificity**	**PPV**	**NPV**	**Diagnostic accuracy**
Lakhkar *et al.*[16]	44.40%	81.80%	80%	47.30%	-
Gaikwad *et al.*[19]	34.92%	91.94%	81.48%	58.16%	63.20%
Present study	32.65%	91.11%	80.00%	55.41%	60.64%

**Table 11:** Comparison of MCS- S/D with other study

**Author**	**Sensitivity**	**Specificity**	**PPV**	**NPV**	**Accuracy**
Biswas *et al.*[20]					
24-30weeks	50%	93%	87.50%	75%	77.77%
31-36weeks	87.50%	83%	65%	94.80%	84.16%
Khanduri *et al.*[21]					
23-27weeks	33.30%	73.90%	68.40%	39.50%	48.40%
≥30 weeks	46.20%	78.30%	78.30%	46.20%	59.10%
Singh *et al.*[22]	86.60%	85%	89.60%	80.90%	86%
Present study	42.86%	93.33%	87.50%	60.00%	67.02%

**Table 12:** Comparison of MCA-RI with other study

**Various Study**	**Sensitivity**	**Specificity**	**PPV**	**NPV**	**Accuracy**
Sachin *et al.* [21]					
23-27weeks	35.90%	82.60%	77.80%	43.20%	53.20%
> 30 weeks	43.60%	87%	85%	47.60%	59.70%
Singh *et al.* [22]	80%	95%	96%	76%	86%
Present study	4.08%	100%	100%	48.91%	50.00%

**Table 13:** Comparison of MCA-PI with other studies

**Various Study**	**Sensitivity**	**Specificity**	**PPV**	**NPV**	**Diagnostic accuracy**
Yash *et al.* [23]	76%	78%	-	-	52%
Kumbar *et al.* [24]	78.90%	68.40%	65.20%	76.40%	70%
Netam *et al.* [17]	47.06%	81.81%	57.14%	75%	70%
Bano *et al.* [25]	8.90%	100%	100%	-	-
Khanduri *et al.*[26]	35.70%	92.60%	91.80%	38.20%	-
Present study	18.37%	97.78%	90.00%	52.38%	56.38%
